# Digestive Profiles of Human Milk, Recombinant Human and Bovine Lactoferrin: Comparing the Retained Intact Protein and Peptide Release

**DOI:** 10.3390/nu16142360

**Published:** 2024-07-21

**Authors:** Bum Jin Kim, Russell F. Kuhfeld, Joanna L. Haas, Yanisa M. Anaya, Raysa Rosario Martinez, Baidya Nath P. Sah, Bella Breen, Kahler Newsham, Carrie-Anne Malinczak, David C. Dallas

**Affiliations:** 1 Nutrition Program, School of Nutrition and Public Health, College of Health, Oregon State University, Corvallis, OR 97331, USA; bumjin.kim@oregonstate.edu (B.J.K.); russell.kuhfeld@oregonstate.edu (R.F.K.); baidyasah@gmail.com (B.N.P.S.); 2 Department of Food Science and Technology, Oregon State University, Corvallis, OR 97331, USA; joanna.haas@oregonstate.edu; 3 Helaina, New York, NY 10010, USA; yanisa@myhelaina.com (Y.M.A.); raysa@myhelaina.com (R.R.M.); isabella@myhelaina.com (B.B.); kahler@myhelaina.com (K.N.); carrie@myhelaina.com (C.-A.M.)

**Keywords:** lactotransferrin, in vitro digestion, peptidomics, protein quantitation, breastmilk, cow’s milk

## Abstract

Lactoferrin (LF) is a major component of human milk. LF supplementation (currently bovine) supports the immune system and helps maintain iron homeostasis in adults. No recombinant human lactoferrin (rhLF) is available for commercial food use. To determine the extent to which rhLF (Effera™) produced by *Komagataella phaffii* digests similarly to hmLF, a validated in vitro digestion protocol was carried out. Bovine LF (bLF) was used as an additional control, as it is approved for use in various food categories. This study compared the extent of intact protein retention and the profile of peptides released in hmLF, bLF and rhLF (each with low and high iron saturation) across simulated adult gastric and intestinal digestion using gel electrophoresis, ELISA and LC-MS. Intact LF retention across digestion was similar across LF types, but the highest iron-saturated hmLF had greater retention in the simulated gastric fluid than all other sample types. Peptides identified in digested hmLF samples strongly correlated with digested rhLF samples (0.86 < r < 0.92 in the gastric phase and 0.63 < r < 0.70 in the intestinal phase), whereas digested bLF samples were significantly different. These findings support the potential for rhLF as a food ingredient for human consumption.

## 1. Introduction

Lactoferrin (LF) is an approximately 80 kDa glycoprotein present in human milk (comprising 10–15% of the total proteins) and various mucosal secretions, such as saliva and intestinal fluids [1]. Human milk LF (hmLF) can bind iron in the gut, which limits the growth of pathogenic microorganisms and enhances iron absorption [2], interacts with the immune system [3] and directly inhibits (unrelated to iron binding) microbial and viral pathogens [4]. Although LF can be purified from human milk, as well as purchased in limited quantities as a research reagent, it is not currently available commercially for supplementation due to both limitations in large-scale production from human donor milk and safety concerns about potential pathogen transmission that would require additional processing to prevent [5].

Bovine LF (bLF) extracted from bovine milk is generally recognized as safe (GRAS) (as per GRAS Notices (GRN) No. 67, 77, 130, 423, 464, 465 and 669) and is used commercially as a nutritional supplement alone or added to foods (e.g., infant formula). Bovine and human milk LF consist of 689 and 691 amino acids (excluding the signal sequence), respectively, and have approximately 70% sequence homology [6]. The 3-D structures of bLF and hmLF are not identical but highly similar [7]. bLF has some in vitro activities that are similar to hmLF: it can bind iron [8], interact with the immune system [1] and inhibit pathogens [9,10]. However, several studies have demonstrated that bLF differs from hmLF in the extent to which it binds to the LF receptor in human intestinal brush border membranes [11], stimulates cytokines in intestinal cell culture models [12], exhibits antimicrobial and antibiofilm effects [13], acts as a prebiotic [14] and alters osteoblast proliferation [15], likely due to the structural differences between bLF and hmLF. More studies directly comparing the effects of bLF and hmLF are needed.

As an alternative source to bLF, recombinant human LF (rhLF) derived from transgenic rice and cows was evaluated as a food ingredient and went through the GRAS notification program in the mid-2000s (GRNs 162 (Lactoferrin (human) purified from rice), 189 (Lactoferrin (human) purified from bovine milk) and 235 (Lactoferrin (human) purified from rice)). Unlike some other recombinant proteins that completed GRAS notification successfully (GRN 1056 for bovine β-lactoglobulin, GRN 737 for soy leghemoglobin and GRN 967 for soluble egg white protein), neither cow nor rice-derived rhLF were permitted for commercial use as a food ingredient due to incomplete safety analysis of its effect on the immune system (e.g., potential for alloimmunization and immunotoxicity) [16]. However, as rhLF can be identical to hmLF in terms of amino acid sequence and highly similar in structure [3], rhLF may have more similar digestive profiles to hmLF than bLF, which could allow it to exert more similar functionality within the human gastrointestinal tract. However, research is needed to compare this digestive profile and its functional impacts.

Both intact LF and LF-derived peptides released across digestion have bioactive functions. Bioactive peptides in human and animal milk have been reported for decades, and a comprehensive database of these peptides was recently compiled [17,18]. Peptides derived from hmLF and bLf have an array of functions, including antimicrobial (69 known peptides), angiotensin-converting enzyme (ACE)-inhibitory (23 known peptides), dipeptidyl peptidase-IV-inhibitory (8 known peptides) and 10 other functional categories. The functional impact of LF depends on which of its forms are present across the digestive tract (and whether any of these forms are absorbed). The extent of similarity and difference in bioactive peptides released during hmLF, rhLF and bLF digestion has not been investigated. Though recombinant forms can have identical amino acid sequences to their native form, differences in their post-translational modifications (e.g., glycosylation) related to the expression system could alter their digestive pattern, resulting in different survival of intact proteins, total peptide generation and the release of bioactive peptides. Thus, further investigation is needed to determine the extent to which the peptides released during the digestion of hmLF differ from those of rhLF and bLF.

The presence or absence of iron alters the tertiary structure of lactoferrin, and the degree of iron saturation can affect susceptibility to protease digestion. Brines et al. [19] reported that iron-saturated bLF and hmLF have more resistance to trypsin and chymotrypsin digestion compared with iron-free LF forms. However, due to the lack of systematic studies on protein and peptide changes of rhLF in the human digestion system, it is unknown how the iron saturation level influences the retention of the intact rhLF and release of bioactive peptides across digestion. Therefore, the extent to which recombinant LF’s digestion (including survival of the intact protein and released peptides) aligns with that of native forms may or may not depend on the degree of iron saturation. As the iron saturation of rhLF can be controlled by altering culture conditions, it is necessary to determine the appropriate iron saturation to best match the digestive profile of hmLF so the information can be used to guide production.

This study aimed to compare the extent to which LF remains intact, as well as the profile of peptides released (including bioactive peptides), among hmLF, rhLF and bLF throughout a validated in vitro simulation of adult digestion via gel electrophoresis, enzyme-linked immunosorbent assay (ELISA) and liquid chromatography–mass spectrometry (LC-MS) analysis. Additionally, the effects of iron saturation on changes of intact LF and peptides across digestion were investigated for each type of LF.

## 2. Materials and Methods

### 2.1. Lactoferrin Sample Acquisition

Helaina Inc. (New York City, NY, USA) produces rhLF (Effera™, α-isoform) by precision fermentation at an industrial scale using the recombinant system of *Komagataella phaffii* [20], a yeast commonly used to make commercial food ingredients. The protein was purified by 0.2 µm microfiltration, 30 kDa ultrafiltration/diafiltration, cation exchange chromatography and then spray-dried to a powder. The purity of the powder was measured by high-performance liquid chromatography (HPLC) and sodium dodecyl sulfate–polyacrylamide gel electrophoresis (SDS-PAGE) and was typically greater than 95% [20]. The iron saturation was determined to be 44% saturated (method measuring the iron saturation described below). Helaina rhLF was reconstituted and diluted to 20 mg/mL with 1× phosphate buffered saline (PBS, pH 7.4, Thermo Fisher Scientific, Waltham, MA, USA) prior to use in the assay.

Donor human milk was collected from 6 donors (maternal age, race and lactation stage not specified) by the Northwest Mothers Milk Bank and pooled and frozen at −20 °C prior to use. The pooled donor human milk was thawed at 25 °C for 1–2 h and hmLF was isolated using the following procedure adapted from Boseman-Finkelstein and Finkelstein [21]: seventeen milk samples (100 mL) were centrifuged at 10,000× *g* for 30 min, the liquid fraction (infranatant) was collected and the fat layer discarded. The resultant liquid fraction was adjusted to pH 4.7 using 1 M hydrochloride (Sigma-Aldrich, St. Louis, MO, USA) before incubation in a bead bath at 40 °C for 30 min for casein precipitation. The solution was then centrifuged at 10,000× *g* for 30 min at 4 °C to separate the supernatant from the precipitated casein.

The supernatant was decanted and purified via ultrafiltration/diafiltration using a 30 kDa molecular weight cut-off filter (MWCO, TangenX PRO pD Casette [Repligen, Waltham, MA, USA]) and cation exchange chromatography (KrosFlo KR2i TFF [Repligen]) with a HiTrap SP HP Column (5 mL, Cytiva, Marlborough, MA, USA) and AKTA sample pump (Cytiva). The column was equilibrated in 50 mM sodium phosphate (pH 7.5, MilliporeSigma, Burlington, MA, USA), and the sample was applied to the column. The column was then washed with 50 mM sodium phosphate (pH 7.5) for five column volumes before being eluted with 50 mM sodium phosphate and 1 M sodium chloride (pH 7.5, MilliporeSigma) via an elution gradient (0 to 100% 1 M sodium chloride) for 20 column volumes. The purified product was then simultaneously buffer-exchanged and concentrated with a 30 kDa MWCO filter (15 mL of filter volume, MilliporeSigma). The sample was concentrated between 1 and 5 mL and filled with 1× PBS (pH 7.4) to the maximum spin filter volume. This process was repeated twice before the sample was concentrated to the final volume. This procedure yielded approximately one gram of LF per liter of human milk with a purity of greater than 95% as measured by HPLC and SDS-PAGE as described previously [20]. The hmLF was diluted to 20 mg/mL with 1× PBS (pH 7.4) prior to use in the assay, and the measured iron saturation was 30% (method for measuring the iron saturation described below).

Bovine LF (bLF) isolated from bovine milk was purchased from Lactoferrin Co, Australia (Product 11683, Lactoferrin Co, Brisbane, Australia) and reconstituted to 20 mg/mL using 1× PBS prior to use. Its measured iron saturation was 6.2%.

The iron content of the LF samples was measured using the AOAC-2015.01 method [22]. Iron saturation was calculated with the following equation: Iron saturation (%) = 2 × (Fe (moles) ÷ lactoferrin (moles)). Fe (moles) was the measured iron content converted to moles (56 Da), and lactoferrin (moles) was the measured LF content (determined by HPLC) converted to moles (80,000 Da).

### 2.2. Iron Saturation Process

hmLF, rhLF and bLF samples at higher levels of iron saturation were prepared. Each LF was reconstituted at 70 mg/mL in 50 mM tris hydrochloride (MilliporeSigma) and 150 mM sodium chloride (pH 7.4, MilliporeSigma), as well as incubated with 1.7 mM ferric nitrate salt (MilliporeSigma) in the presence of 1.7 mM nitrilotriacetic acid (MilliporeSigma) at a molar ratio of 1:2:2 = LF–ferric nitrate–nitrilotriacetic acid with mixing by stir bar and stir plate at room temperature (20 °C) for 2 h. After incubation, excess iron was removed by dialysis (10 kDa MWCO, Slide-A-Lyzer dialysis cassettes, Thermo Fisher Scientific) against 50 mM sodium phosphate (pH 5.6) for 36 h with three water changes. Each protein that underwent iron saturation was concentrated and buffer-exchanged into 1× PBS (pH 7.4) using the spin columns (30 kDa MWCO, Sigma-Aldrich) described previously. All samples were diluted to 20 mg/mL using 1× PBS (pH 7.4). The iron content of each protein type after the iron saturation process was determined via the method described above.

### 2.3. INFOGEST Static In Vitro Digestion of Lactoferrin Samples

**Figure 1:**
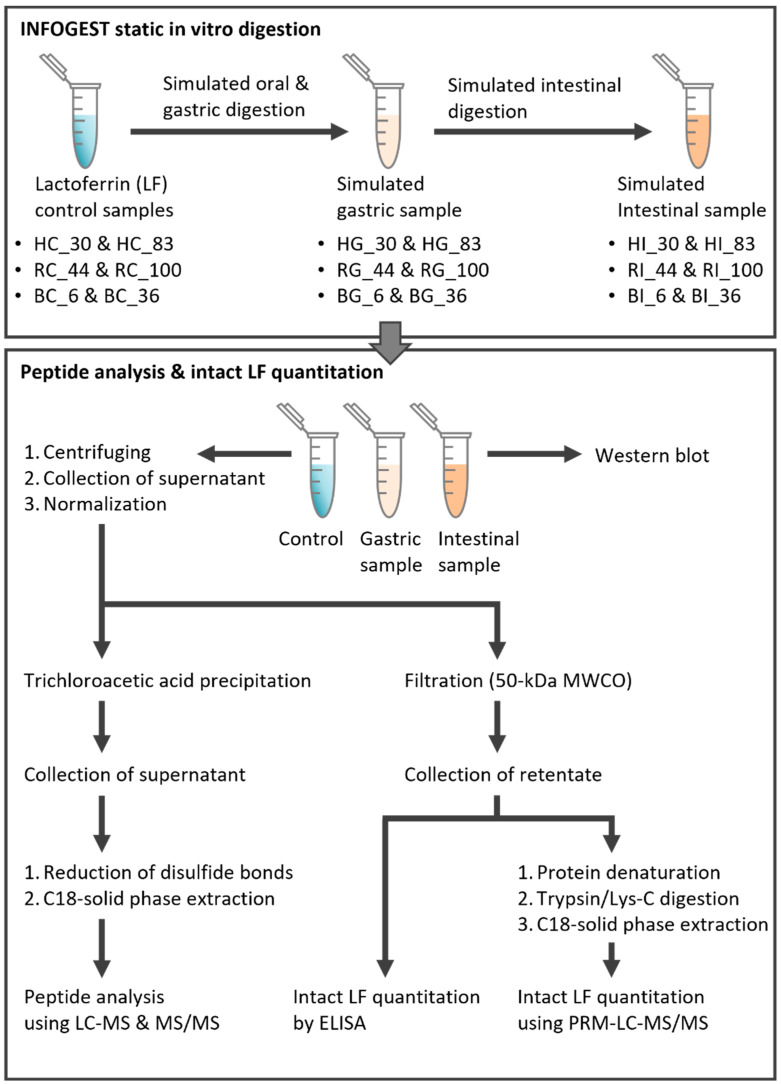
Experimental workflow for peptide analysis and intact protein quantitation of human, bovine and recombinant human lactoferrin across simulated digestion (INFOGEST static in vitro simulation). Sample names indicate LF type (H, human milk; R, recombinant human; and B, bovine), digestion phase (C, control; G, gastric; and I, intestinal) and iron saturation level (%).

An in vitro simulation of adult digestion was carried out on all samples (Figure 1). This digestion included subsequent incubations of each sample with simulated salivary, gastric and intestinal fluid prepared as specified in the INFOGEST protocol [23]. LF stocks were prepared in advance, aliquoted and stored at −20 °C until ready for use. The in-house method deviated from the INFOGEST protocol in that an automated pH titrator was not used, so the components of each simulated digestive fluid were not added in the exact order specified. The digestive enzymes were added last after making pH adjustments so that the zero-time point would represent the time the enzymes interacted with the lactoferrin instead of the time the fluid was titrated to the correct pH. As the analysis focused on the digestion profile of protein only, α-amylase and lipase were omitted in the simulated salivary and gastric fluids, respectively. After two minutes of salivary phase digestion, 500 μL of the samples were collected, split into 100 μL aliquots and stored at −80 °C. After 120 min of gastric digestion, 5 mL of the samples were collected, and the pepsin was immediately inactivated with 0.2 mL of sodium bicarbonate (pH 11). The gastric sample was split into aliquots that were immediately stored at −80 °C. After 120 min of intestinal digestion, 4 mL of intestinal fluid was collected and split into 2 mL aliquots each, with protease inhibiter (5 mM Pefabloc, MilliporeSigma) added in one set for intact protein quantitation and one set without for peptidomic analysis. All samples were then stored at −80 °C.

### 2.4. Confirmation of LF Digestion via Gel Electrophoresis and Western Blot

All samples were initially assessed via SDS-PAGE. More specifically, all experimental samples were run neat, and each LF stock control sample was diluted with 1× PBS to a concentration of 0.1 mg/mL in 16 μL of total sample volume, according to the manufacturer’s protocol for 4–12% Tris-Glycine SDS-PAGE (Thermo Fisher Scientific). Two microliters of protein standard were added to the first lane of each gel. The gels were placed in a mini gel tank and run at 185 V for 45 min. They were then removed from their cassettes and placed in an opaque gel box on a rocker (VWR, Radnor, PA, USA) at medium speed and tilt. The gels were rinsed with deionized water for 5 min and then stained with Imperial Protein Stain (Thermo Fisher Scientific) for 45 min. They were then destained overnight with deionized water and imaged the following morning with a gel imaging system (iBright imager, Thermo Fisher Scientific).

Western blot using anti-LF antibodies was conducted to visualize the retained intact human LF throughout the digestion in the control and digestive samples following the procedure described previously in Anaya et al. [24]. The control was diluted to 0.025 mg/mL (136-fold) with 1× PBS, and the simulated gastric and intestinal fluid samples were run neat. The SDS-PAGE gels were also prepared as described above, but after removal from the gel tank, they were transferred onto a PVDF membrane using a gel transfer device (iBlot 2, Thermo Fisher Scientific). The membrane was incubated in blocking solution (1× PBST + 10% Fish Gelatin [Biotium 10× Fish Gelatin Blocking agent] and 10× PBS, Thermo Fisher Scientific) on a rocker at room temperature for 1 h. After washing, the primary antibody (LF antibody, diluted 5000-fold, MilliporeSigma, Burlington, MA, USA) was added and incubated for 1 h. The membrane was then washed and incubated in a secondary antibody (Goat anti-Rabbit IgG [H+L], Alexa Fluor™ 488, 2000-fold, Thermo Fisher Scientific) for 30 min. Following the final incubation, protein samples were visualized with fluorescent imaging using excitation at 455–485 nm and emission at 508–557 nm (iBright imager, Thermo Fisher Scientific).

### 2.5. Extraction of the Intact LF

The control and the digestive samples were centrifuged at 12,000× *g* at 4 °C for 30 min to precipitate the solid debris, and the liquid layers (including the intact lactoferrin) were collected (Figure 1). Intact LF was extracted from peptides and co-existing small molecules in the samples via molecular weight cut-off filtration. Centrifugal 50 kDa MWCO filter devices (0.5 mL of filter volume, MilliporeSigma, Burlington, MA, USA) were rinsed with 500 μL of ultrapure water at 12,000× *g* at 4 °C for 10 min. This step was repeated two more times. Aliquots of control (7.35 μL) and digestive samples (15.29 μL of gastric and 30.96 μL of intestinal) were mixed with ultrapure water to a final volume of 500 μL, added to each rinsed filter and then centrifuged at 12,000× *g* at 4 °C for 10 min. Then, the retentate, including the intact LF in the filter, was collected using a pipette and dried by vacuum centrifugation. Dried intact LF in each sample was reconstituted with 1 mL of ultrapure water for quantification by ELISA and LC-MS analysis. Extracted LF samples were stored at −80 °C until analysis.

### 2.6. Determination of Intact LF Concentration by ELISA

The concentration of the intact LF in each sample was analyzed using bovine (ab200015, Abcam, Cambridge, UK) and human (ab274406, Abcam) ELISA kits (Figure 1). Prior to analysis, all samples were diluted in the dilution buffer (as provided by the manufacturer) to ensure that their optical density values fell within the linear range of the assays. Native bLF samples (control, gastric and intestinal) were diluted 250-fold, and native hmLF and rhLF controls were diluted 40,000-fold, whereas gastric and intestinal hmLF and rhLF samples were diluted 10,000-fold. Following dilution, samples were measured in duplicate according to the ELISA kit protocols provided by the manufacturer. The optical density was measured at 450 nm using a spectrometer (SpectraMax M2 microplate reader, Molecular Devices, San Jose, CA, USA). Lactoferrin concentrations were calculated from the quadratic calibration curve created via software (SoftMax Pro 7.0, Molecular Devices). The relative concentration of LF (%) for each sample was calculated by the following equation: measured concentration of each sample ÷ measured concentration of the control sample × 100.

To assess potential matrix interference, detection efficiency was calculated for both assays based on the method outlined by Liang et al. [25]. Exactly 250 μL of each diluted bLF sample was spiked with 50 ng/mL bLF by adding 5 μL of a 2550 ng/mL bLF standard. Similarly, 250 μL of diluted hmLF and rhLF samples were spiked with 2 ng/mL hmLF by adding 5 μL of a 2000 ng/mL standard.

### 2.7. Quantitation of the Intact LF Using LC-MS/MS

Intact LF extracts in the control and intestinal samples were quantified by parallel-reaction monitoring (PRM) with LC-MS/MS, as described previously with modifications [26] (Figure 1). Briefly, an aliquot of 300 μL of each extracted intact LF sample from the control and intestinal samples was completely dried by vacuum centrifugation, and the dried samples were reconstituted, denatured using dithiothreitol and iodoacetamide and digested using Lys-C/trypsin. Tryptic peptides were purified by C18-solid phase extraction (SPE). In parallel, hmLF and bLF standards were prepared using the same approach to make calibration curves for each type of LF. Unique peptides produced from LF trypsin digestion, SDTSLTWNSVK (*m*/*z* 619.3066, *z* = 2) for hmLF and rhLF and VDSALYLGSR (*m*/*z* 540.7878, *z* = 2) for bLF, were analyzed to quantify each LF using PRM-LC-MS/MS, and data processing was performed using Skyline (MacCoss Lab, University of Washington, Seattle, WA, USA) [27] to make a standard curve and calculate the LF concentration in each sample. Relative concentrations of each sample were calculated as described above in Section 2.6.

### 2.8. Extraction of LF-Derived Peptides in Control and Digestive Samples

Aliquots of control (7.35 μL) and digestive samples (15.29 μL of gastric and 30.96 μL of intestinal) prepared as described in Section 2.5 were mixed with ultrapure water to a final volume of 100 μL (Figure 1). One hundred microliters of 24% trichloroacetic acid (Sigma-Aldrich) was added to each sample and centrifuged at 4000× *g* at 4 °C for 10 min to precipitate the proteins. Supernatants including the peptides were collected in a new tube and dried by vacuum centrifugation. Dried samples were then redissolved with 100 μL of 50 mM ammonium bicarbonate and transferred to a 96-well plate. To reduce the disulfide bonds of the peptides, dithiothreitol and iodoacetamide activation were performed as described previously [28]. Peptides were cleaned by C18-SPE. The C18 96-well plate (50 mg bed weight, 50 μm particle size; Biotage, Uppsala, Sweden) was washed and reconditioned with 900 μL of ultrapure water, 900 μL of 80% acetonitrile (ACN, Thermo Fisher Scientific), 0.1% trifluoroacetic acid (MilliporeSigma) and 900 μL of ultrapure water prior to the loading of the samples using an SPE plate vacuum manifold. After sample loading, the 96-well plate was washed with 900 μL of ultrapure water to remove salts and interfering substances. Then, 900 μL of 80% ACN and 0.1% trifluoroacetic acid was added to elute the peptides. Eluates were transferred to conical tubes and dried by vacuum centrifugation.

### 2.9. Peptide Analysis Using LC-MS/MS with Data-Dependent Acquisition

Peptides extracted from the control and digestive samples were analyzed using an Orbitrap Fusion™ Lumos™ Tribrid™ mass spectrometer (Thermo Fisher Scientific) connected to a nanoACQUITY UPLC (Waters, Milford, MA, USA) as described in the previous study [28] (Figure 1). Briefly, dried samples were reconstituted with 50 μL of 3% ACN, 0.1% formic acid and diluted 5-fold. One microliter of the sample was injected onto a C18 trap column and separated on a C18 column over 60 min. Peptides were ionized with electrospray ionization, and full MS scans were acquired in positive ionization mode in the Orbitrap. Following the full MS scan, precursor ions were fragmented using higher-energy collisional dissociation in the Orbitrap.

Spectral data files were analyzed in Proteome Discoverer (v3.0.1.27, Thermo Fisher Scientific), employing the Sequest HT search engine against human (P02788) and bovine (P24627) lactoferrin protein sequences. The allowed dynamic modifications included the oxidation of methionine, phosphorylation of serine and threonine and acetylation of the N-terminus. The carbamidomethylation of cysteine was set as a fixed modification. Only peptides with high-confidence (threshold: *p <* 0.01) peptide-spectral matches (PSM) were included in the subsequent analysis. The total peptide count, indicating the number of peptides identified in each sample replicate, was obtained from Proteome Discoverer. The abundances of each identified peptide were measured using the area under the curve of the extracted ion chromatograms based on ion intensity.

### 2.10. Statistical Analysis

One-way analysis of variance with post hoc Tukey’s Honest Significant Difference was employed (type 1 error rate = 0.05) using RStudio (Version 2023.06.2+561) to understand the similarities and differences among the samples. For analyses, samples were categorized based on various groupings, including the type of LF (protein type: bLF, hmLF or rhLF), the varied percentage of iron saturation and simulated digestion state (control, gastric or intestinal). Pearson correlation analysis (r) was conducted to compare the abundance of peptides that were identical across different sample types (bLF, hmLF and rhLF at each iron saturation) by data analysis in Excel. Heatmapping of peptide relative abundances was performed using Heatmapper [29] with the following parameters: “Average Linkage” at clustering method and “Pearson” at distance measurement method.

A heatmap overlaying species-specific LF sequences of origin (human/recombinant or bovine) was generated showing the density of the identified peptides. The number of overlapping peptide sequences was summed using Jupyter Notebook and plotted above the respective protein sequence on the *x*-axis, as performed previously [30]. The line graph illustrates the average instrument abundance corresponding to the amino acid sequence of peptides originating from the protein type.

### 2.11. Bioactivity Prediction of LF-Derived Peptides

Identified peptides were examined for homology with literature-identified bioactive peptides using the Milk Bioactive Peptide Database (MBPDB; https://mbpdb.nws.oregonstate.edu/ (accessed on 16 April 2024)) [17,18], which provides each peptide’s biological functions and the associated literature sources. The search parameters were set to “Sequence” as the search type, “80%” as the similarity threshold, “Identity” as the scoring matrix and both “lactotransferrin (P24627)” and “lactotransferrin (P02788)” as the proteins.

## 3. Results

### 3.1. Iron Saturation of the LF Samples

The degree of iron saturation was effectively increased for each of the LF types after the iron saturation procedure (Table 1). Iron saturation varied across proteins at both the initial and higher iron saturation levels.

### 3.2. Quantitation of Intact LF in Gastric and Intestinal Samples

The detection efficiency of all spiked samples in ELISA ranged from approximately 35% to 112% (Supplementary Table S1).

**Figure 2:**
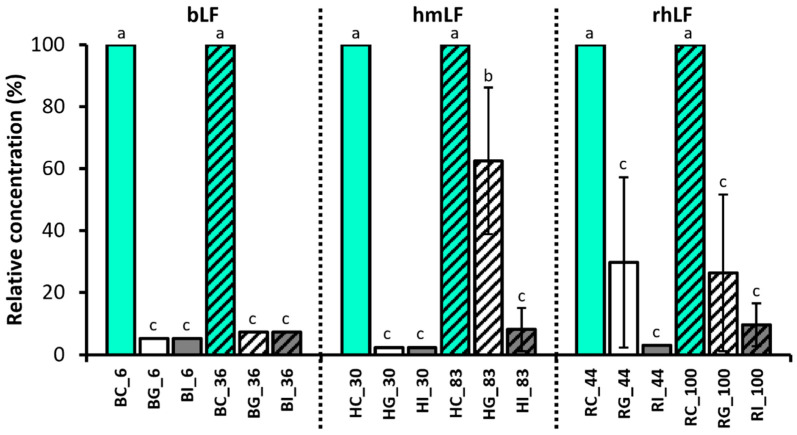
Relative concentration (%) of the intact hmLF, bLF and rhLF measured via ELISA of the digestive samples compared with the control. Relative concentration was calculated with the following equation: measured LF concentration in the digestive sample divided by its concentration in the control sample × 100, and it was averaged and expressed in the bar graph as the mean values ± standard deviation. Solid and pattern bars indicate the low- and high-iron-saturated samples, respectively. Differing letters indicate significant differences in the relative concentration of the intact LF among sample types (Tukey’s HSD). Sample names indicate LF type (H, human milk; R, recombinant human; and B, bovine), digestion phase (C, control; G, gastric; and I, intestinal) and iron saturation level (%).

Based on ELISA, the intact hmLF with lower iron saturation (30%) significantly decreased in percent survival after gastric and intestinal digestion (both HG_30 and HI_30 were below the limit of detection [LOD]; Figure 2 and Supplementary Table S2). Percent survival of the higher iron-saturated hmLF (83%) significantly decreased after the gastric phase (on average 62.5% ± 32.9% standard deviation) and decreased further in the intestinal phase (8.2% ± 7.1%). The percent survival in the gastric phase for 83% iron-saturated hmLF was significantly higher than for 30% iron-saturated hmLF.

For the low-iron-saturation rhLF (44%), the percent survival of LF decreased significantly after the gastric (29.7% ± 30.8%) and intestinal phases (below the LOD) compared with the control but did not differ statistically between gastric and intestinal samples. Similarly, the survival of higher-iron-saturated rhLF (100%) was significantly lower after both gastric (26.4% ± 26.4%) and intestinal phases (9.7% ± 11.9%), which did not differ statistically between the digestive samples. There were no significant differences between the percent survival of rhLF in the gastric or intestinal digests between lower and higher iron saturation.

The relative concentrations of both gastric and intestinal samples of bLF were below the LOD at both iron saturation levels. Therefore, there were no differences in the extent of bLF survival between the lower (6%) and higher (36%) iron saturation levels.

Across all sample types, the percent retention of LF in the gastric sample was significantly higher for hmLF with high iron saturation (83%). The percent survival for LF in the intestinal phase did not differ across sample types.

**Figure 3:**
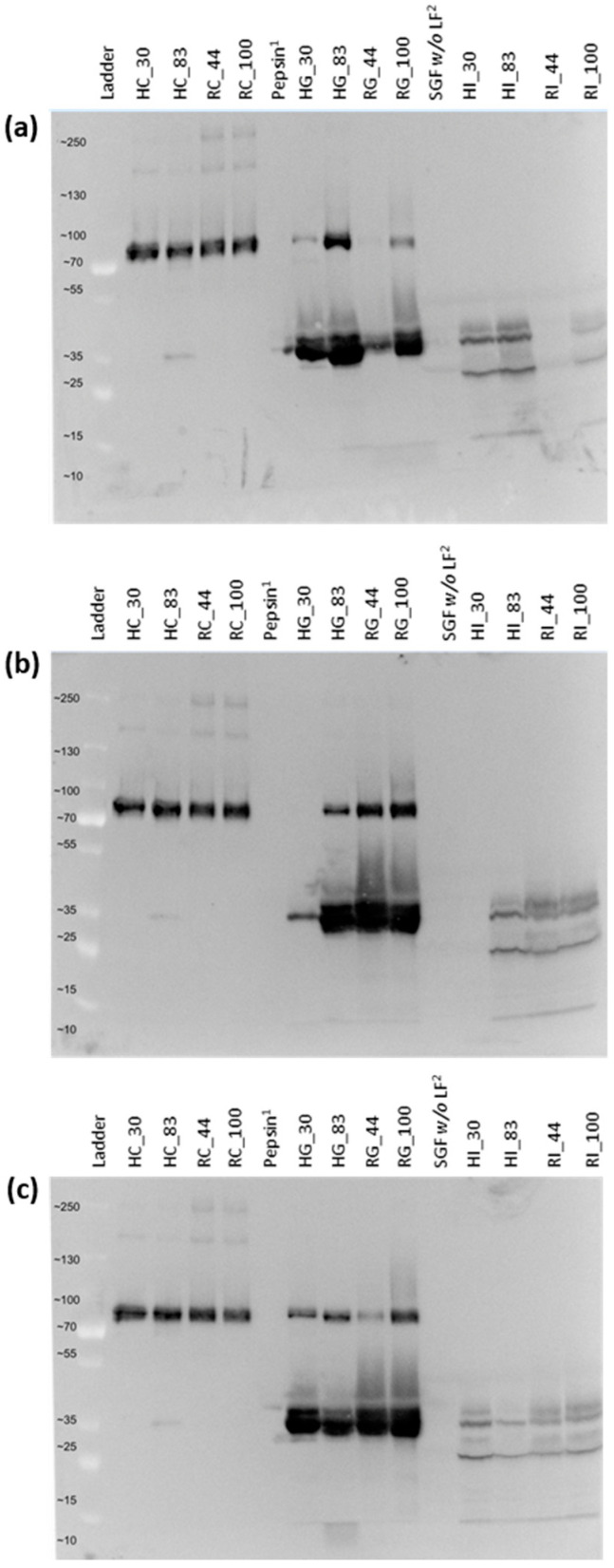
Gel images of the intact lactoferrin (H, hmLF and R, rhLF) and LF-derived peptides in the control (C, no digestion) and digestive samples (G, gastric; and I, intestinal) separated by Western blot. The numerical value at the end of the sample ID represents the iron saturation levels. Western blot was performed in triplicate (**a**–**c**). ^1^ Pepsin denotes the pepsin solution used for gastric digestion. ^2^ SGF *w/o* LF means that the gastric sample was digested without LF.

Gel images of Western blots with anti-LF antibodies show one abundant gel band at 80 kDa (intact LF molecular weight range) in the control and gastric samples (Figure 3). Intact LF was retained in the high-iron-saturated gastric samples (HG_83 and RG_100), and relatively thin gel bands were present in the low-iron-saturated gastric samples (HG_30 and RG_44) compared to the high-iron samples. In the gastric samples, digested LF bands were observed at 25–45 kDa. In the intestinal phase, no intact LF remained for either hmLF or rhLF, regardless of iron saturation level. The intestinal phase showed mostly overlapping peptide bands between 20 and 40 kDa, as well as faint bands between 10 and 15 kDa for hmLF and rhLF.

SDS-PAGE was used to further confirm the findings of the Western blot analysis for hmLF and rhLF and also to visualize bLF survival across digestion (Supplementary Figure S1). The results of the SDS-PAGE gels showed consistent results in the gastric phase of hmLF and rhLF compared to Western blot. Relatively higher abundant gel bands for intact LF were observed in the HG_83 and RG_100 samples than the HG_30 and RG_44 samples. Partially digested LF bands were also observed between 25 and 45 kDa. The SDS-PAGE image did not show intact bLF following gastric digestion (BG_6 and BG_36). The gel band patterns from 25 to 45 kDa for bLF differed from those for hmLF and rhLF. In the gel bands of the intestinal samples, significant visual differences were not observed.

The concentration of the intact LF in many of the intestinal samples (except HI-83 and RI_100), as measured via ELISA, was lower than the LOD. To confirm these findings, the intact LF in the control and intestinal samples were further quantified by PRM-based LC-MS/MS analysis (Supplementary Figure S2). The relative concentrations of the retained intact LF were 2.1% (±0.79%) and 0.76% (±0.09%) in HI_30 and HI_83 and 2.04% (±0.47%) and 6.23% (±1.43%) in RI_44 and RI_100, respectively. The intact bLF concentrations in BI_6 and BI_36 measured by PRM-LC-MS/MS analysis were below the LOD. Thus, no intact bLF was identified in the bovine digestive samples by ELISA, SDS-PAGE or PRM-LC-MS/MS analysis. Both ELISA and Western blot showed consistent results in that they both found that the measured intact LF among the gastric samples was highest in HG_83, followed by RG_44 and RG_100, and was lowest in HG_30. Quantitative analysis of intact hmLF and rhLF in the intestinal phase using ELISA and PRM-LC-MS/MS showed the same quantitation pattern for RI_44 and RI_100 (higher in RI_100), whereas they showed a different pattern for HI_30 and HI_83 (higher in HI_83 by ELISA but higher in HI_30 by PRM-LC-MS/MS).

### 3.3. Peptide Analysis Using LC-MS and MS/MS

#### 3.3.1. Comparison of Peptides between the Control and Digestive Samples

**Figure 4:**
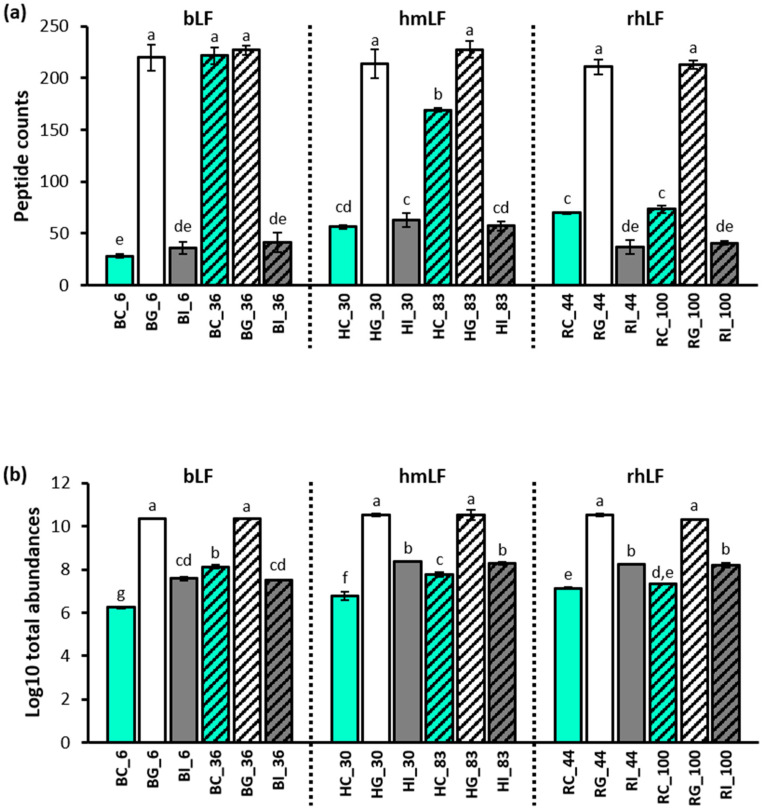
Comparison of the peptides identified in each sample. Bar graph expresses (**a**) counts (the number of identified peptides) and (**b**) abundances (sum of total peak area of all peptides) of bLF, hmLF and rhLF peptides as the mean values ± standard deviation. Solid and pattern bars indicate the low- and high-iron-saturated samples, respectively. Differing letters indicate significant differences in the relative concentration of the intact LF among sample types (Tukey’s HSD). Sample names indicate LF type (H, human milk; R, recombinant human; and B, bovine), digestion phase (C, control; G, gastric; and I, intestinal) and iron saturation level (%).

Peptidomics enabled the identification of peptides present for each LF type at each stage of digestion (Supplementary Tables S3–S5). Peptide counts of the higher-iron-saturated samples were significantly higher in bLF and hmLF control samples (*n* = 222 in BC_36 and 169 in HC_83) than in the lower-iron-saturated samples (*n* = 28 in BC_6 and 56 in HC_36), whereas peptide counts were similar between RC_44 (*n* = 70) and RC_100 (*n* = 73; Figure 4a). In both high- and low-iron-saturated samples across bLF, hmLF and rhLF, the number of peptides in the gastric samples was significantly higher than in the control and intestinal samples (on average, *n* = 220 in BG_6, 214 in HG_30, 228 in HG_83, 211 in RG_44 and 213 in RG_100). bLF at 36% iron saturation increased from control to gastric samples numerically but not significantly (222 in BC_36 and 227 in BG_36). In the peptide analysis and the subsequential data processing, replicate #2 of BG_36 was deemed an outlier and excluded, as it exhibited an anomalous peptide profile compared to all other samples. In the intestinal samples, peptide counts were significantly higher for HI_30 than all bLF and rhLF intestinal samples, but not significantly higher than HI_83. Numerically, peptide counts were highest in hmLF samples (*n* = 63 at HI_30 and 57 at HI_83), followed by rhLF (*n* = 37 at RI_44 and 41 at RI_100) and bLF (*n* = 36 at BI_6 and 41 at BI_36). Relative peak area values (log10) of the total peptides identified in gastric samples were more abundant than in the control and the intestinal samples for all protein types (Figure 4b). Total peptide abundances in intestinal samples were higher than in the paired control samples except the high-iron-saturated bLF sample (36%).

#### 3.3.2. Comparison of Peptides between the Low- and High-Iron-Saturated Samples

**Figure 5:**
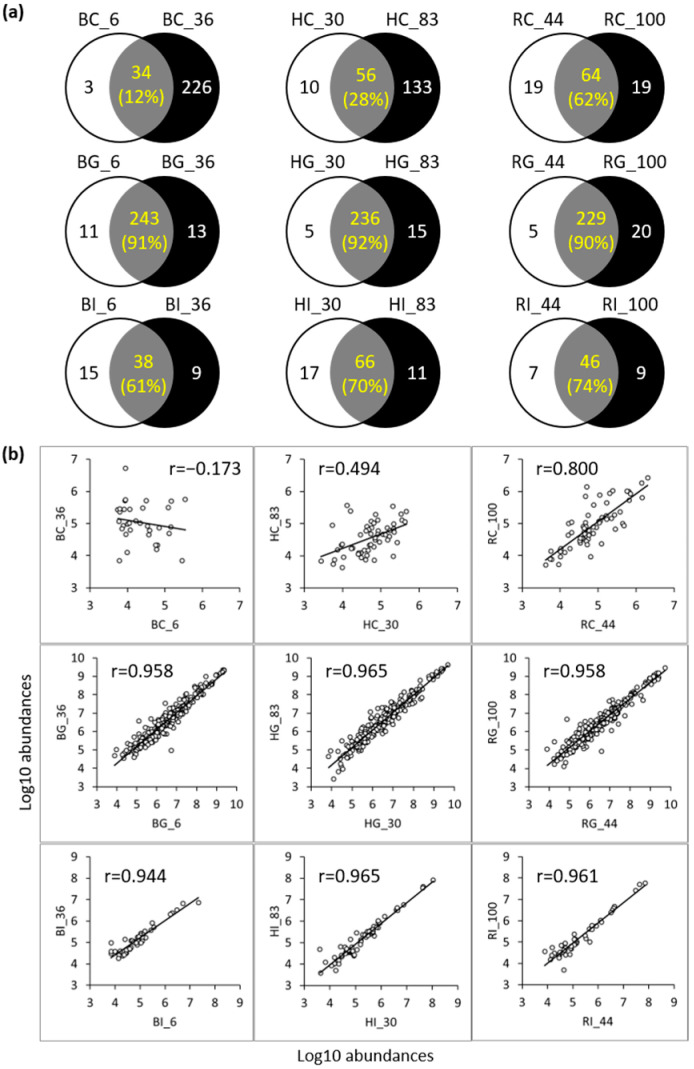
Comparison of the peptides identified in the control and digestive samples before and after iron saturation. (**a**) Venn diagram expresses the counts of the identified peptides in low- and high-iron-saturated samples, and (**b**) scatter plots show the similarities/differences between the peptides that were identified in both samples. The peptide correlations between the samples were evaluated by correlation analysis using peptide abundances expressed with the log10 scale. The Pearson correlation coefficient (r) is displayed at the top of the graph. Sample names indicate LF type (H, human milk; R, recombinant human; and B, bovine), digestion phase (C, control; G, gastric; and I, intestinal) and iron saturation level (%). Numbers under the peptide counts identified in common indicate the percentage of those counts that were identified as in common as calculated by the following equation: Count of the peptides in common ÷ (Counts of the unique peptides in each sample + Count of the peptides in common) × 100.

The number of peptides that were the same between the low- and high-iron-saturated forms of bLF were 34 in the control sample, 243 in the gastric sample and 38 in the intestinal samples (Figure 5a). Similarly, over 200 peptides were identified in both low- and high-iron-saturated gastric samples of hmLF (HG_30 and HG_83) and rhLF (RG_44 and RG_100), and fewer peptides were identified in common in control and intestinal samples of hmLF and rhLF (Figure 5a). Low- and high-iron-saturated rhLF had 62% identical peptides in the control sample, 90% identical peptides in the gastric phase and 74% identical peptides in the intestinal phase. Low- and high-iron-saturated hmLF had 28%, 92% and 70% identical peptides for control, gastric and intestinal samples, respectively. Low- and high-iron-saturated bLF had 12%, 91% and 61% identical peptides for control, gastric and intestinal samples, respectively. In general, the largest percentage of identical peptides for each was in the gastric phase, followed by the intestinal phase, and the control samples had the most variation. The peptides that were identical between the low- and high-iron-saturated forms of each protein type were examined to determine the extent to which the abundances of these peptides correlated with the low- and high-iron-saturated protein digesta (Figure 5b). The abundances of peptides found in both low- and high-iron-saturated forms correlated highly between low- and high-iron-saturated bLF, hmLF and rhLF in both the gastric and intestinal phases (0.944 < r < 0.965; Figure 5b). For control samples, rhLF had relatively lower correlation coefficients (RC_44 vs. RC_100, r = 0.800), and bovine (BC_6 vs. BC_36, r = −0.173) and human (HC_30 vs. HC_83, r = 0.494) samples had poor peptide profile correlations.

#### 3.3.3. Comparison of Peptides between the hmLF and rhLF or bLF Samples

**Figure 6:**
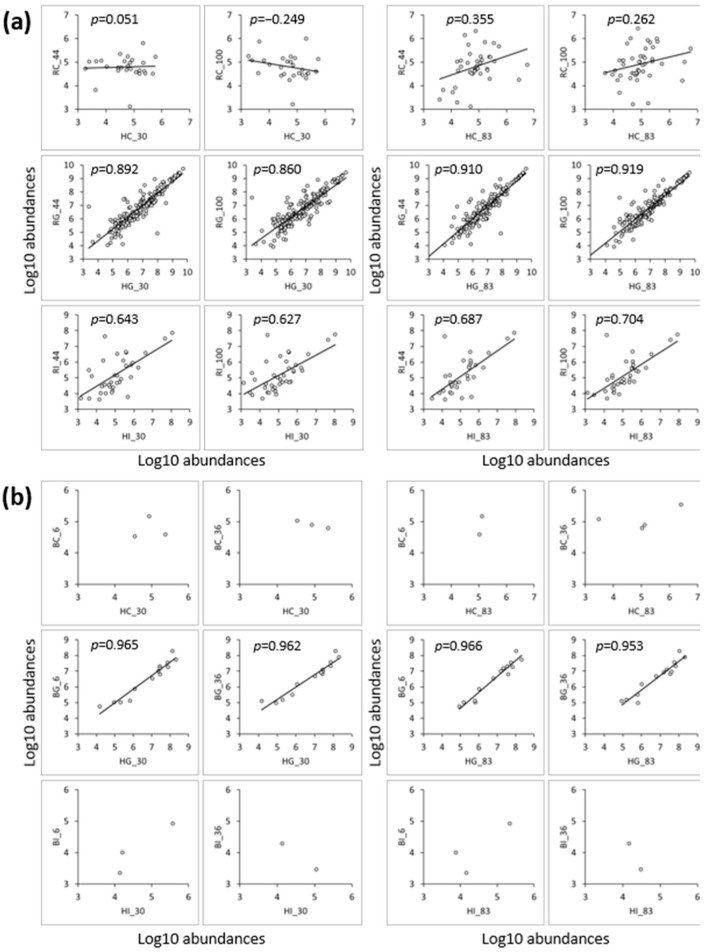
Comparison of the peptides identified in both hmLF and (**a**) rhLF or (**b**) bLF samples. Scatter plots show the similarities/differences between the peptides that were identified in both samples. The peptide correlations between the samples were evaluated by correlation analysis using peptide abundances expressed with the log10 scale. The Pearson correlation coefficient (r) is displayed at the top of the graph. Sample names indicate LF type (H, human milk; R, recombinant human; and B, bovine), digestion phase (C, control; G, gastric; and I, intestinal) and iron saturation level (%).

The peptides identified in hmLF were compared with those in rhLF or bLF samples. A higher percentage of the total peptides were identified in common between hmLF and rhLF (16–55% in common) than between hmLF and bLF (≤3% in common; Supplementary Figure S3). Correlation analysis was conducted to examine similarities in each of the identical peptide’s abundances across hmLF and rhLF or bLF samples (Figure 6). Peptide profiles between gastric hmLF and rhLF samples were highly similar (0.860 < r < 0.919), whereas the similarities between intestinal hmLF and rhLF samples were relatively lower (0.627 < r < 0.704). Control samples of hmLF and rhLF exhibited the lowest similarity in their peptide profiles (0.051 < r < 0.355). For hmLF and bLF samples, correlation analysis could not be performed for the control (HC and BC) and intestinal (HI and BI) samples due to their low number of common peptides (*n* = 2–4). Peptide profiles identified in both gastric samples (HG and BG) showed similar patterns, but only 14 peptides were identified in common, and over 200 peptides were present in each sample uniquely.

**Figure 7:**
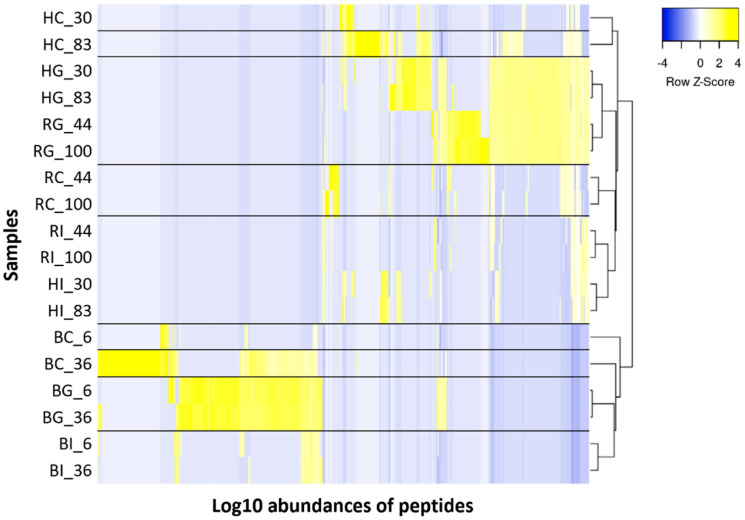
Heatmap and hierarchical clustering express similarities and differences between the peptide abundances (log10 scale) identified in each sample. Sample names indicate LF type (H, human milk; R, recombinant human; and B, bovine), digestion phase (C, control; G, gastric; and I, intestinal) and iron saturation level (%).

In the heat maps with hierarchical clustering, control samples of hmLF, rhLF and bLF were clustered with each other and separated from the digestive samples (Figure 7). Gastric or intestinal samples of hmLF and rhLF samples had similar peptide abundance patterns, and each digestion type was clustered in the same group. Digestive samples of bLF were more different from hmLF and rhLF than hmLF and rhLF were from each other (based on hierarchical clustering).

Counts and abundances of peptides identified in each sample (combining both low- and high-iron-saturated and control, gastric and intestinal phases) were summarized and visualized across the LF sequence (Supplementary Figure S4), and the results showed that peptides from hmLF and rhLF had more similar patterns compared with bLF. For instance, in regions 287–312, bLF exhibited a lower peptide abundance and total count compared with hmLf and rhLF.

### 3.4. Investigation of Bioactive Peptides

Peptides with homology to known bioactive peptides were identified in hmLF, rhLF and bLF control and digestive samples via MBPDB search (Supplementary Tables S6–S8). Two bioactive peptides with 100% homology to known bioactive peptides (RYYGY and KYLGPQY) were identified in both hmLF and rhLF, both of which have opioid antagonist activity [31]. RYYGY was present in all sample types (control, gastric and intestinal) for both hmLF and rhLF. KYLGPQY was present in all gastric samples, most control samples and one intestinal sample across hmLF and rhLF. bLF had three bioactive peptides (YLGSRY, YLTTLK and FKSETKNLL) with 100% homology to known bioactivities, and none of these peptides were present in hmLF and rhLF digests. YLGSRY, known as a lactoferroxin A, is an opioid antagonist peptide [32], YLTTLK is an antiviral peptide [33] and FKSETKNLL has osteoanabolic properties [34].

**Figure 8:**
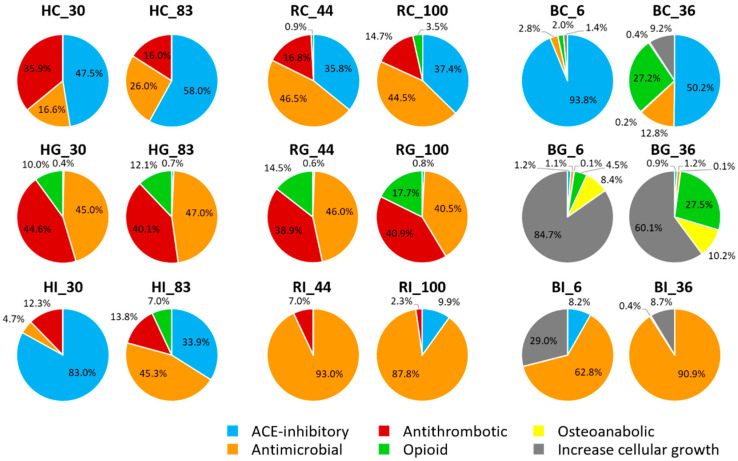
Comparison of relative abundances (%) of bioactive peptides. Bioactivity of the identified peptides in each sample was predicted via MBPDB search, and all matched peptides were grouped based on their assigned bioactive functions (blue, ACE-inhibitory; orange, antimicrobial; red, antithrombotic; green, opioid; yellow, osteoanabolic; and gray, increase cellular growth). Sample names indicate LF type (H, human milk; R, recombinant human; and B, bovine), digestion phase (C, control; G, gastric; and I, intestinal) and iron saturation level (%).

The number of peptides identified with >80% homology to known bioactive peptides ranged from 3 to 17 across sample types and digestive states (Table 2). For all protein types, the number of potentially bioactive peptides found in gastric samples was higher than in the control and intestinal samples. Bioactive peptide counts of rhLF samples were similar to those of hmLF samples (across both digestion phase and iron saturation level), whereas bLF samples had higher counts compared to both hmLF and rhLF samples. The percentages of relative abundances of the identified bioactive peptides grouped based on the matched bioactive functions were calculated and compared (Figure 8). In the control samples, ACE-inhibitory function was predominant in hmLF and bLF samples, whereas antimicrobial function was most abundant in rhLF samples. Peptides with antimicrobial and opioid functions (including four peptides from hmLF and five peptides from rhLF that are highly homologous with the known antimicrobial peptide human lactoferrampin (AA 287-303, WNLLRQAQEKFGKDKSP [35])) were highly abundant in hmLF and rhLF gastric samples. Peptides relating to cellular growth function were dominant in bLF gastric samples. We identified three peptides homologous with bovine lactoferrampin (AA 287-303, WKLLSKAQEKFGKNKSR [36]) in the bLF gastric digest. In the intestinal samples, antimicrobial function was highest in all LF samples except the low-iron-saturated hmLF sample (HI_30, ACE-inhibitory was highest). In the intestine, we also identified one peptide in the hmLF and two peptides in the rhLF samples that were homologous with human lactoferrampin. In the bLF intestinal digestion, we identified two peptides that were homologous with bovine lactoferrampin. Peptides with >80% homology with human or bovine lactoferricin were not identified in any sample.

## 4. Discussion

### 4.1. Quantitation of the Intact LF in Control and Digestive Samples

Intact LF retained in the simulated digestive samples was measured via gel electrophoresis, ELISA and PRM-LC-MS quantitation.

The Western blots, SDS-PAGE and ELISA analysis indicate some degree of survival of hmLF and rhLF across gastric digestion, followed by nearly complete degradation after intestinal digestion. These results align with those of past studies that found that LF derived from human milk had some degree of resistance to in vitro gastric digestion but not intestinal digestion. For example, Inglingstad et al. [37] reported that around 33% of LF from raw human milk survived intact across ex vivo digestion with adult human gastric juices at pH 2.5 for 30 min but was completely lost after digestion with human duodenal juices at pH 8.0 for 30 min (measured via SDS-PAGE). For infant digestion, Sánchez-Hernández et al. [38] found over 66% survival of hmLF after simulated infant in vitro gastric digestion (pH 5.3, 1 h) and complete loss of hmLF after the intestinal phase (pH 6.6, 1 h). Elwakiel et al. [39] found, via gel electrophoresis and LC-MS, a similar degree of survival of hmLF from human colostrum and mature milk after simulated infant in vitro gastric (pH 5, 1 h, 33% survival for colostrum, 12% for mature) and intestinal digestion (pH 7.0, 1 h, 1% survival for colostrum, 3% for mature).

The measured concentrations of bLF were below the LOD in the digestive samples in the ELISA and PRM-LC-MS (intestinal only) results (Figure 2 and Figure S2), and none of the gel images showed bands for intact LF at around 80 kDa in SDS-PAGE (Supplementary Figure S1). Most of the previous studies reported that bLF was completely digested in the in vitro gastric digestion and did not reach the simulated intestinal phase in the intact form [40,41,42,43]. Bokkhim et al. [44], however, found that more than half of the bLF survived intact across in vitro gastric digestion for 2 h via SDS-PAGE. A potential reason for the discrepancy in results is that Bokkhim et al. conducted the in vitro digestion with lower enzyme activity (180 U pepsin/mg LF) across the gastric digestion compared to the current study (1600 U pepsin/mg LF), and this difference might have affected the extent of the bLF that remained intact in the gastric phase. Bokkhim et al. found nearly complete degradation of bLF during intestinal digestion, which aligns with our results. Furlund et al. [45] also reported that intact bLF was not detectable via SDS-PAGE across either gastric digestion (pH 2.5, 30 min) or intestinal digestion (pH 7.0, 30 min), whereas, after gastric digestion at a higher pH (pH 4.0, 30 min), abundant gel bands of intact bLF were observed in both gastric and intestinal samples. In the current study, simulated adult in vitro digestion was performed at pH 3.0 for 2 h for the gastric phase and pH 7.0 for 2 h for intestinal phase, resulting in the significant digestion of the intact bLF across in vitro digestion.

This study revealed that intact hmLF and rhLF retention were more similar to each other than hmLF and bLF retention were. hmLF and rhLF were retained intact at relatively higher levels than bLF across gastric digestion.

The retention of intact LF notably differed between low- (30%) and high- (83%) iron-saturated hmLF in the gastric phase, indicating that iron saturation could influence the digestibility of hmLF. Though several studies have reported that the degree of iron saturation can affect the extent of digestion of bLF [19,44,46], there is a lack of past research examining the effect of the degree of iron saturation on intact hmLF survival across gastrointestinal digestion. One study investigated the resistance of proteolysis by trypsin and chymotrypsin between native and iron-saturated hmLF samples (iron saturation percentages not provided) via SDS-PAGE [19]. Both trypsin and chymotrypsin digests (activated for 3 h) resulted in an appreciable amount of intact hmLF remaining regardless of the iron saturation level [18]. However, the present study is the first to reveal that high iron saturation can increase survival across adult gastric digestion for hmLF compared with low iron saturation.

There were no clear differences observed in the survival of low- and high-iron-saturated forms for rhLF in the ELISA data. However, the Western blot data suggest the higher gastric survival of intact rhLF (100% iron saturation) compared with rhLF (44% iron saturation) in two of three replicates.

The measured concentration of the retained intact hmLF and rhLF in ELISA and the observed gel bands of intact hmLF and rhLF in the gastric samples showed a high variation among the replicates (Figure 2 and Figure 3). This discrepancy in the gastric samples suggests variability between triplicates due to the complex nature of simulated digestion.

### 4.2. Peptide Analysis in Control and Digestive Samples

Overall, the patterns of counts of peptides released were similar across bLF, hmLF and rhLF, with counts increasing from the control to the gastric phase and decreasing from the gastric to the intestinal phase (Figure 4a). Similarly, for bLF, hmLF and rhLF across low and high iron saturation, peptide abundance increased from the control to the gastric phase and decreased from the gastric to the intestinal phase (Figure 4b).

Comparing each protein across low and high iron saturation revealed no significant differences in total peptide counts and total peptide abundances between low- and high-iron-saturated hmLF, rhLF or bLF across digestion (Figure 5). Comparisons between low and high iron saturation for each protein present revealed a large degree of overlap in specific peptides between each set at each digestive phase. Most peptides identified were identical for the gastric and intestinal phases of the low- and high-iron-saturated forms of each protein. Among these peptides that were identical, the abundances of each peptide were highly correlated for the gastric and intestinal phases of the low- and high-iron-saturated forms of each protein. These findings indicate that altering the degree of iron saturation for each of these proteins had little impact on which peptides were released in the gastric and intestinal phases and at what abundances they were released. To our knowledge, no previous study has investigated the effect of iron saturation on the extent of peptide release across digestion in either hmLF or bLF.

Comparisons of the peptide profiles of hmLF to rhLF and bLF indicate a much larger degree of similarity between hmLF and rhLF than hmLF and bLF, regardless of iron saturation levels (Figure 6 and Figure 7). Correlation analysis revealed a highly strong correlation (0.919 ≥ r ≥ 0.627) between the peptidomic profiles of hmLF and rhLF in the gastric and intestinal samples. Gel electrophoresis results also supported that the rate of digestion, as well as the peptides generated, was substantially similar between hmLF and rhLF (Figure 3 and Figure S1). The percentages of peptides that were identical across hmLF and rhLF were much higher than those across hmLF and bLF. Of the hundreds of peptides identified in the digestive bLF and hmLF samples, only 14 peptides in the gastric phase and 2 to 3 peptides in the intestinal phase were found in common. Correlation analysis was not completed for the intestinal phase for bLF and hmLF as they had so few overlapping peptides. A larger number of overlapping peptides were present in the stomach for bLF and hmLF, though they represented a small percentage of the total peptides identified. Among this small portion of the overall peptides, there was, interestingly, a strong correlation (0.966 ≥ r ≥ 0.953) between peptide abundances across protein types. The greater peptide profile overlap between hmLF and rhLF than hmLF and bLF is likely related to the fact that hmLF and rhLF have 100% amino acid sequence homology, whereas hmLF and bLF have only 71% homology [6]. Higher sequence homology makes identifying the same peptides across samples much more likely. Moreover, changes in the amino acid sequence can affect cleavage patterns for digestive proteases. Also, sequence differences can affect protein secondary and tertiary structures, which can impact the accessibility of cleavage sites for digestive proteases.

In addition to differences in amino acid sequence between hmLF and bLF, differences in glycosylation patterns may also contribute to the dissimilarity in their peptide profiles. In hmLF, complex/hybrid types of N-glycans consisting of 3–6 Hex, 3–7 HexNAc, 0–3 Fuc and 0–2 NeuAc are mainly present (>99%) on three N-glycosylation sites at Asn156, Asn497 and Asn642 [47]. bLF differs from hmLF in that high mannose-type N-glycans are most abundant (65–84%) in bLF, followed by the complex/hybrid type (16–35%), which attach to 5 N-glycosylation sites at Asn252, Asn300, Asn387, Asn495 and Asn564 [48,49,50]. Moreover, the complex/hybrid-type N-glycans on bLF contain epitopes that do not exist in hmLF, including NeuGc, GalNAc-GalNAc (LacdiNAc) and Gal-Gal-GlcNAc (αGal) [51]. The differences in N-glycosylation patterns between bLF and hmLF may alter enzyme activity and cleavage site accessibility across the gastrointestinal digestion, partly explaining the differences in peptide releases in digestion between hmLF and bLF.

rhLF N-glycan compositions vary based on the source of rhLF production. For example, Fujiyama et al. [52] reported that rice-based rhLF N-glycans contained Xyl and α1-3-Fuc, which are absent from human glycans, and a lack of NeuAc, which is present in human glycans. Site-specific profiling of N-glycans in rhLF produced in *K. phaffii* (the rhLF used in the present study) indicated that N-glycans were attached to the same N-glycosylation sites (Asn156, Asn497 and Asn642) as in hmLF, but differed in glycan composition, as it was dominated by large mannosylated N-glycans (41%) and high mannose-type glycans (59%) rather than the complex/hybrid types dominating hmLF [20]. Compared to bLF, rhLF peptide profiles in the digestive samples exhibited a higher similarity to hmLF. The similarities in the sites of N-glycosylation between hmLF and rhLF likely contributed to the similarity of their peptide profiles across gastric and intestinal digestion. Though rhLF and hmLF had highly similar peptide profiles, they showed some differences. Differences in the N-glycan compositions of rhLF compared to those of hmLF may have contributed to these observed differences. However, another potential source for the differences observed between rhLF and hmLF include that the hmLF used herein was derived from a pool of milks, which could include some proportion of hmLF with single nucleotide polymorphisms that lead to amino acid changes in the expressed lactoferrin protein [53].

### 4.3. Investigation of Bioactive Peptides of bLF, hmLF and rhLF across Digestion

hmLF and rhLF digests contained the same two known bioactive peptides, RYYGY and KYLGPQY. These peptides have opioid antagonist activity [31]. The presence of opioid antagonists in the gut could modulate digestive processes. The bioactive peptides present in digests of bLF (three peptides: YLGSRY, YLTTLK and FKSETKNLL) were completely different from those present in hmLF and rhLF. Lactoferroxin A (YLGSRY) is an opioid antagonist peptide [32], YLTTLK is an antiviral peptide that inhibits rotavirus infection [33] and FKSETKNLL can regulate the proliferation and differentiation of osteoblasts [34].

Beyond examining exactly matched bioactive peptides (100% homology), we decided to assess which peptides had >80% homology to known bioactive peptides because we wanted to identify peptides that are likely bioactive. In general, the number of potential bioactive peptides (>80% homology) increased from control to the gastric samples and then decreased in the intestinal samples. This pattern strongly suggests that the gastric phase plays a pivotal role in the release of bioactive peptides during digestion. Among the identified bioactive peptides, 14 peptides were found in both hmLF and rhLF samples, whereas only 1 peptide (KLRPVA) was present in both the hmLF and bLF samples. We also found a greater overlap of functionality of bioactive peptides in the hmLF and rhLF gastric digests than in the hmLF and bLF (Table 2 and Figure 8). Both hmLF and rhLF had mostly antimicrobial and opioid functions, whereas bLF had mostly cellular growth-related peptides. In the intestinal phase, neither rhLF nor bLF with hmLF had a very high functional overlap of peptides.

bLF digests had a larger number of potential bioactive peptides released (17) than the hmLF and rhLF samples did (7 and 9, respectively; Table 2). Part of the reason for this increased number of potential bioactive peptides from bLF is that this protein has been studied more extensively for bioactive peptides than hmLF, which increases the likelihood of finding overlaps with known peptides (63 bioactive peptides have been found from bLF in the literature according to the MBPDB, whereas 35 peptides have been found for hmLF [18]).

## 5. Conclusions

Overall, rhLF (Effera™, α-isoform) was observed to be highly similar to hmLF in terms of intact protein survival and peptide release throughout simulated adult digestion. We observed that iron saturation impacted gastric digestion for hmLF intact survival (based on both ELISA and Western blot data), that it may have impacted intact rhLF during gastric digestion (based on Western blot data but not ELISA data) and that it did not affect intact bLF (based on both ELISA and SDS-PAGE data). Peptide release profiles across digestion did not appear to be majorly impacted by iron saturation for any of the proteins. Further investigation using more comparable iron saturation levels could lead to a better understanding of the relationship between iron saturation level and LF digestion. Our findings indicate that the rhLF tested had significantly higher similarity to hmLF than bLF in terms of intact LF survival rate and released peptide profiles. We observed the presence of known bioactive peptides and potential bioactive peptides from LF across all protein types and digestive states. The predicted bioactive peptide profiles for hmLF were more similar to rhLF than those of bLF.

The much larger degree of similarity of the digestive profiles between the tested rhLF and hmLF than between hmLF and bLF indicates that supplementation with rhLF could better mimic the effects of hmLF than bLF. However, additional research examining the use of rhLF is needed, including (1) an evaluation of safety; (2) comparison of rhLF and hmLF changes across in vivo digestion; and (3) comparison of the physiological activity of rhLF and hmLF glycosylated and non-glycosylated peptides across digestion.

## Figures and Tables

**Table 1:** Information about human milk and recombinant human and bovine lactoferrin samples.

Sample Name ^1^	Lactoferrin Type	Digestion Stage	Iron Saturation (%)
HC_30	Human milk (native)	Control	29.9
HG_30		Gastric	
HI_30		Intestinal	
HC_83		Control	82.5
HG_83		Gastric	
HI_83		Intestinal	
RC_44	Recombinant human	Control	43.7
RG_44		Gastric	
RI_44		Intestinal	
RC_100		Control	100.0
RG_100		Gastric	
RI_100		Intestinal	
BC_6	Bovine (native)	Control	6.2
BG_6		Gastric	
BI_6		Intestinal	
BC_36		Control	35.9
BG_36		Gastric	
BI_36		Intestinal	

^1^ The first letter of the sample ID represents the lactoferrin type: H, human milk; R, recombinant human; and B, bovine. The second letter of the sample ID represents the digestion stage: C, control (no digestion); G, gastric; and I, intestinal. The ending number of sample ID represents the percentage of iron saturation.

**Table 2:** Summary of the bioactive peptides identified in each lactoferrin sample via MBPDB search.

	Sample Name ^1^					
	HC_30	HG_30	HI_30	HC_83	HG_83	HI_83
Counts of peptides ^2^	5	7	5	5	7	5
Counts of bioactive functions ^3^						
ACE-inhibitory	2	1	1	2	2	1
Antimicrobial	2	12	3	2	12	1
Opioid	2	4	2	2	4	3
Osteoanabolic	0	3	0	0	3	1
	RC_44	RG_44	RI_44	RC_100	RG_100	RI_100
Counts of peptides	8	7	3	8	9	5
Counts of bioactive functions						
ACE-inhibitory	3	2	0	3	4	1
Antimicrobial	5	14	3	3	14	6
Opioid	3	3	1	2	3	1
Osteoanabolic	1	3	0	1	3	0
	BC_6	BG_6	BI_6	BC_36	BG_36	BI_36
Counts of peptides	5	17	4	14	17	5
Counts of bioactive functions						
ACE-inhibitory	2	8	1	7	8	0
Antimicrobial	1	14	6	11	14	7
Antithrombotic	0	1	0	1	1	0
Opioid	1	4	0	4	4	2
Osteoanabolic	0	5	0	2	5	0
Increase cellular growth	2	6	3	9	5	2

^1^ The first letter of the sample ID represents the lactoferrin type: H, human milk; R, recombinant human; and B, bovine. The second letter of the sample ID represents the digestion stage: C, control (no digestion); G, gastric; and I, intestinal. The ending number of the sample ID represents the percentage of iron saturation. ^2^ Counts denote the number of peptides assigned from the database. ^3^ Counts denote the number of input peptides matched with the peptide list in the database. Some peptides matched with multiple (more than two) database peptides and/or bioactive functions.

## Data Availability

The original contributions presented in the study are included in the article and supplementary material.

## Supplementary Materials

The following supporting information can be downloaded at https://www.mdpi.com/article/10.3390/nu16142360/s1, Figure S1: SDS-PAGE separation of the intact lactoferrin (H, human milk; R, recombinant human; and B, bovine) and LF-derived peptides in the control (C, no digestion) and digestive samples (G, gastric; and I, intestinal). The numerical value at the end of the sample ID represents the iron saturation levels. SDS-PAGE was performed in triplicate (a, b and c). Negative controls are the samples digested without LF via simulated in vitro intestinal digestion. Figure S2: The relative concentration of the intact LF in the intestinal samples measured by PRM-LC-MS/MS quantitation of each type of sample. The relative concentrations of the intact LF in the intestinal samples were calculated against the absolute concentration measured in each control sample. Solid and pattern bars indicate the low- and high-iron-saturated samples, respectively. Differing letters indicate significant differences in the relative concentration of the intact LF among sample types (Tukey’s HSD). Sample names indicate LF type (H, human milk; R, recombinant human; and B, bovine), digestion phase (C, control; G, gastric; and I, intestinal) and iron saturation level (%). Figure S3: Venn diagram shows the identified peptides in hmLF and either rhLF or bLF samples. Sample names indicate LF type (H, human milk; R, recombinant human; and B, bovine), digestion phase (C, control; G, gastric; and I, intestinal) and iron saturation level (%). Numbers under the peptide counts identified in common indicate the percentage of those counts in common calculated by the following equation: Count of the peptides in common ÷ (Counts of the unique peptides in each sample + Count of the peptides in common) × 100. Figure S4: This line graph depicts average abundances of peptides present in all samples (combining both low- and high-iron-saturated and control, gastric and intestinal phases) grouped by the different LF types (bLF, blue line; hmLF, green line; and rhLF, red line). The heat map below the x-axis of the line graph indicates the site-specific density of the peptide counts present at each position in the amino acid sequence. On the heat map, red color indicates a high number of overlapping peptide sequences, with decreasing numbers illustrated by orange and then yellow shading. Green indicates a lack of known peptides in that portion of the protein sequences. Table S1. Detection efficiency assessment across human milk, recombinant and bovine lactoferrin samples via ELISA. Table S2. Measured intact lactoferrin concentrations and calculated relative concentrations of human milk, recombinant and bovine lactoferrin samples. Table S3. List of the peptides identified in human milk lactoferrin samples. Table S4. List of the peptides identified in recombinant human lactoferrin samples. Table S5. List of the peptides identified in bovine lactoferrin samples. Table S6. List of the bioactive peptides identified in human milk lactoferrin samples via MBPDB search. Table S7. List of the bioactive peptides identified in recombinant human lactoferrin samples via MBPDB search. Table S8. List of the bioactive peptides identified in bovine lactoferrin samples via MBPDB search.

## Abbreviations

bLF, bovine lactoferrin; ELISA, enzyme-linked immunosorbent assay; hmLF, human milk lactoferrin; LC-MS, liquid chromatography–mass spectrometry; LF, lactoferrin; rhLF, recombinant human lactoferrin; MBPDB, Milk Bioactive Peptide Database; MWCO, molecular weight cut-off; SDS-PAGE, sodium dodecyl-sulfate polyacrylamide gel electrophoresis; SPE, solid phase extraction.
