# Supplementary material for: Digestive Profiles of Human Milk, Recombinant Human and Bovine Lactoferrin: Comparing the Retained Intact Protein and Peptide Release
Source: Nutrients. 2024 Jul 21;16(14):2360. doi: 10.3390/nu16142360 (PMC11280017; doi:10.3390/nu16142360)
Supplement: Supplementary file 1 [file nutrients-16-02360-s001.zip › Supplementary figures.pptx]

## Slide 1
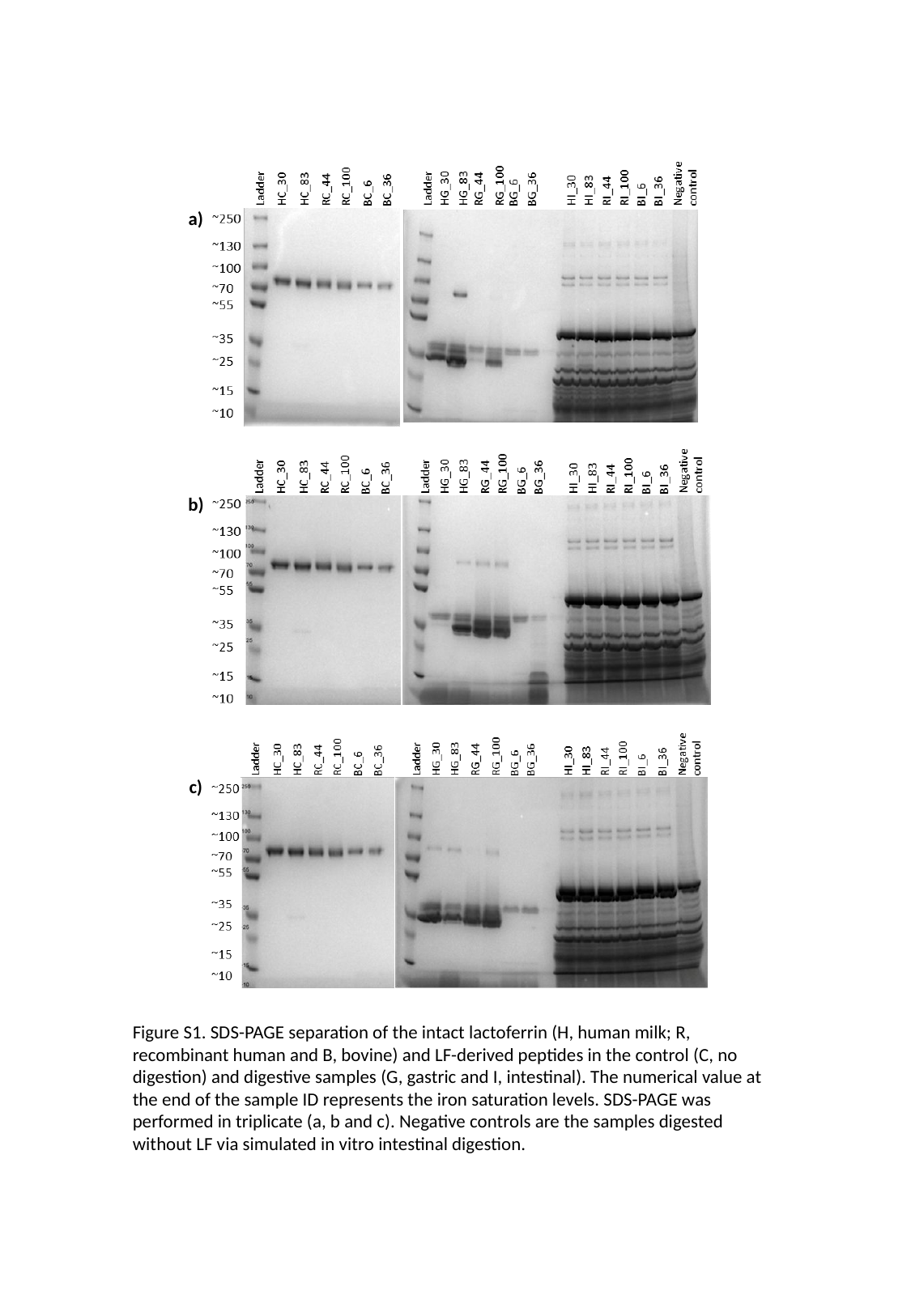

a)
b)
c)
Figure S1. SDS-PAGE separation of the intact lactoferrin (H, human milk; R, recombinant human and B, bovine) and LF-derived peptides in the control (C, no digestion) and digestive samples (G, gastric and I, intestinal). The numerical value at the end of the sample ID represents the iron saturation levels. SDS-PAGE was performed in triplicate (a, b and c). Negative controls are the samples digested without LF via simulated in vitro intestinal digestion.

## Slide 2
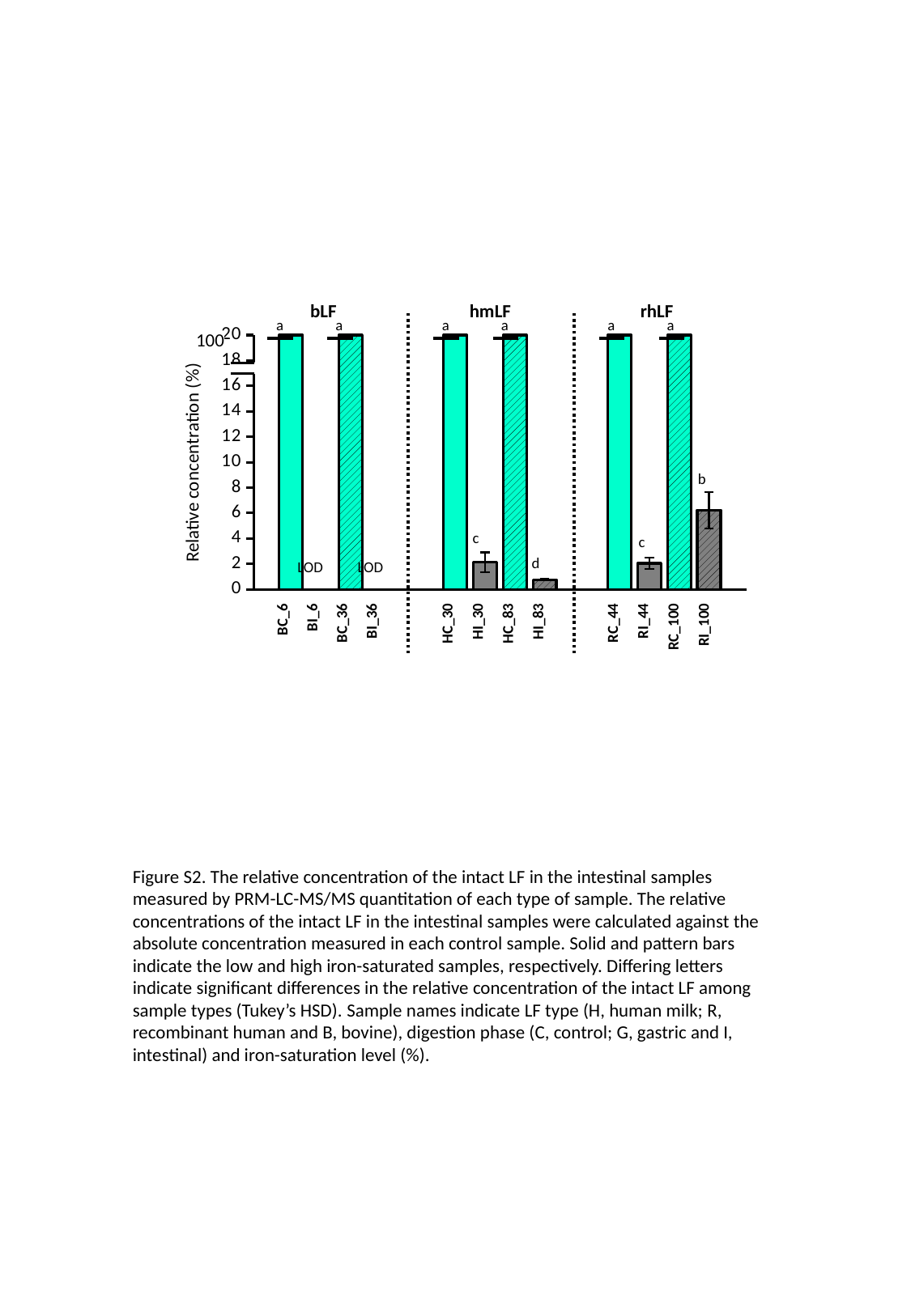

bLF
hmLF
rhLF
a
a
a
a
a
a
### Chart
| Category | | | | |
|---|---|---|---|---|
| bLF | 100.0 | 0.0 | 100.0 | 0.0 |
| hLF | 100.0 | 2.116217270075868 | 100.0 | 0.7645979772307108 |
| rhLF | 100.0 | 2.0431545518104066 | 100.0 | 6.234837399997734 |100
LOD
LOD
b
c
c
d
BC_6
BI_6
BC_36
BI_36
HC_30
HI_30
HC_83
HI_83
RC_44
RI_44
RC_100
RI_100
Figure S2. The relative concentration of the intact LF in the intestinal samples measured by PRM-LC-MS/MS quantitation of each type of sample. The relative concentrations of the intact LF in the intestinal samples were calculated against the absolute concentration measured in each control sample. Solid and pattern bars indicate the low and high iron-saturated samples, respectively. Differing letters indicate significant differences in the relative concentration of the intact LF among sample types (Tukey’s HSD). Sample names indicate LF type (H, human milk; R, recombinant human and B, bovine), digestion phase (C, control; G, gastric and I, intestinal) and iron-saturation level (%).

## Slide 3
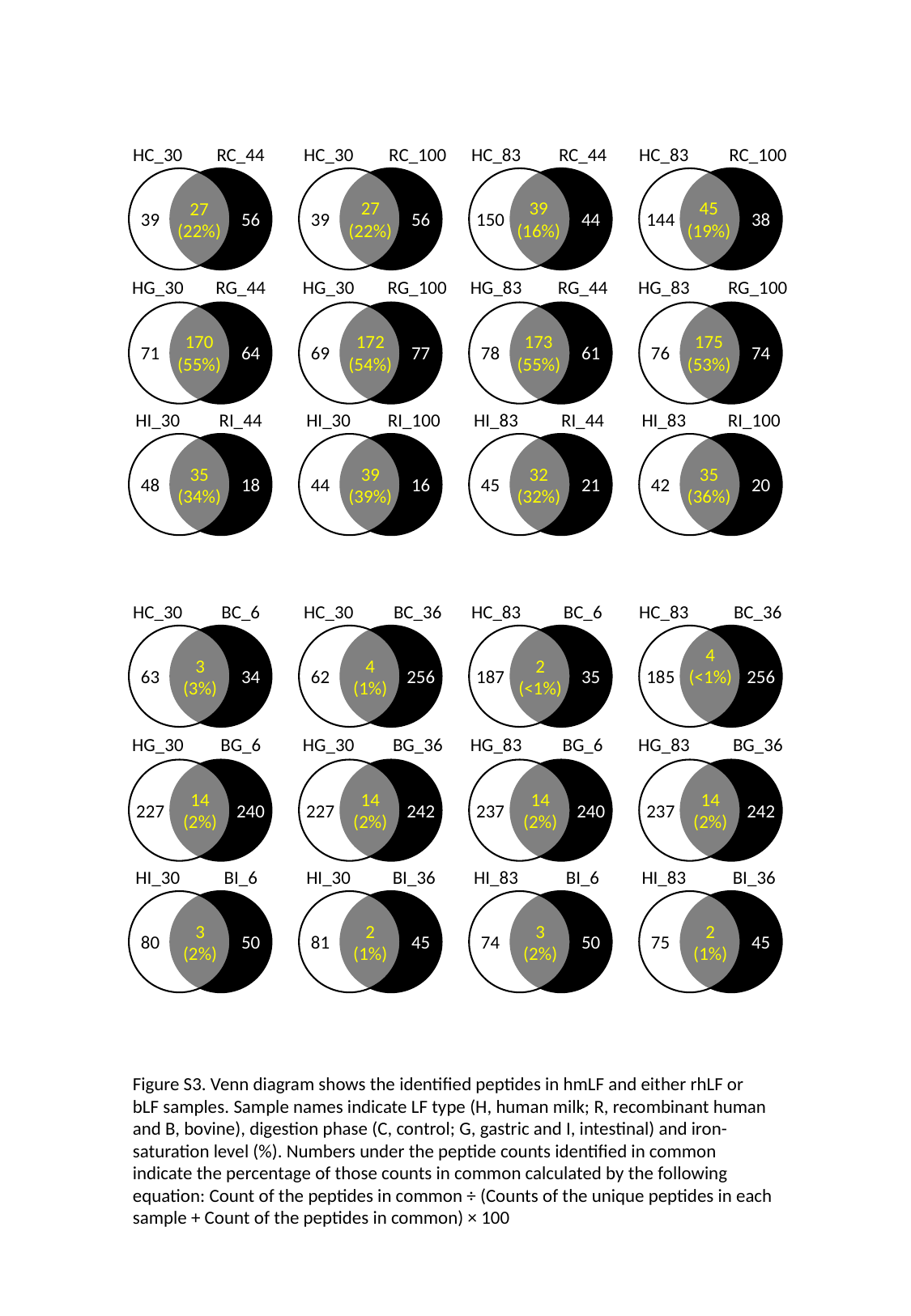

HC_30
RC_44
HC_30
RC_100
HC_83
RC_44
HC_83
RC_100
27
(22%)
27
(22%)
39
(16%)
45
(19%)
39
56
39
56
150
44
144
38
HG_30
RG_44
HG_30
RG_100
HG_83
RG_44
HG_83
RG_100
170
(55%)
172
(54%)
173
(55%)
175
(53%)
71
64
69
77
78
61
76
74
HI_30
RI_44
HI_30
RI_100
HI_83
RI_44
HI_83
RI_100
35
(34%)
39
(39%)
32
(32%)
35
(36%)
48
18
44
16
45
21
42
20
HC_30
BC_6
HC_30
BC_36
HC_83
BC_6
HC_83
BC_36
4
(<1%)
3
(3%)
4
(1%)
2
(<1%)
63
34
62
256
187
35
185
256
HG_30
BG_6
HG_30
BG_36
HG_83
BG_6
HG_83
BG_36
14
(2%)
14
(2%)
14
(2%)
14
(2%)
227
240
227
242
237
240
237
242
HI_30
BI_6
HI_30
BI_36
HI_83
BI_6
HI_83
BI_36
3
(2%)
2
(1%)
3
(2%)
2
(1%)
80
50
81
45
74
50
75
45
Figure S3. Venn diagram shows the identified peptides in hmLF and either rhLF or bLF samples. Sample names indicate LF type (H, human milk; R, recombinant human and B, bovine), digestion phase (C, control; G, gastric and I, intestinal) and iron-saturation level (%). Numbers under the peptide counts identified in common indicate the percentage of those counts in common calculated by the following equation: Count of the peptides in common ÷ (Counts of the unique peptides in each sample + Count of the peptides in common) × 100

## Slide 4
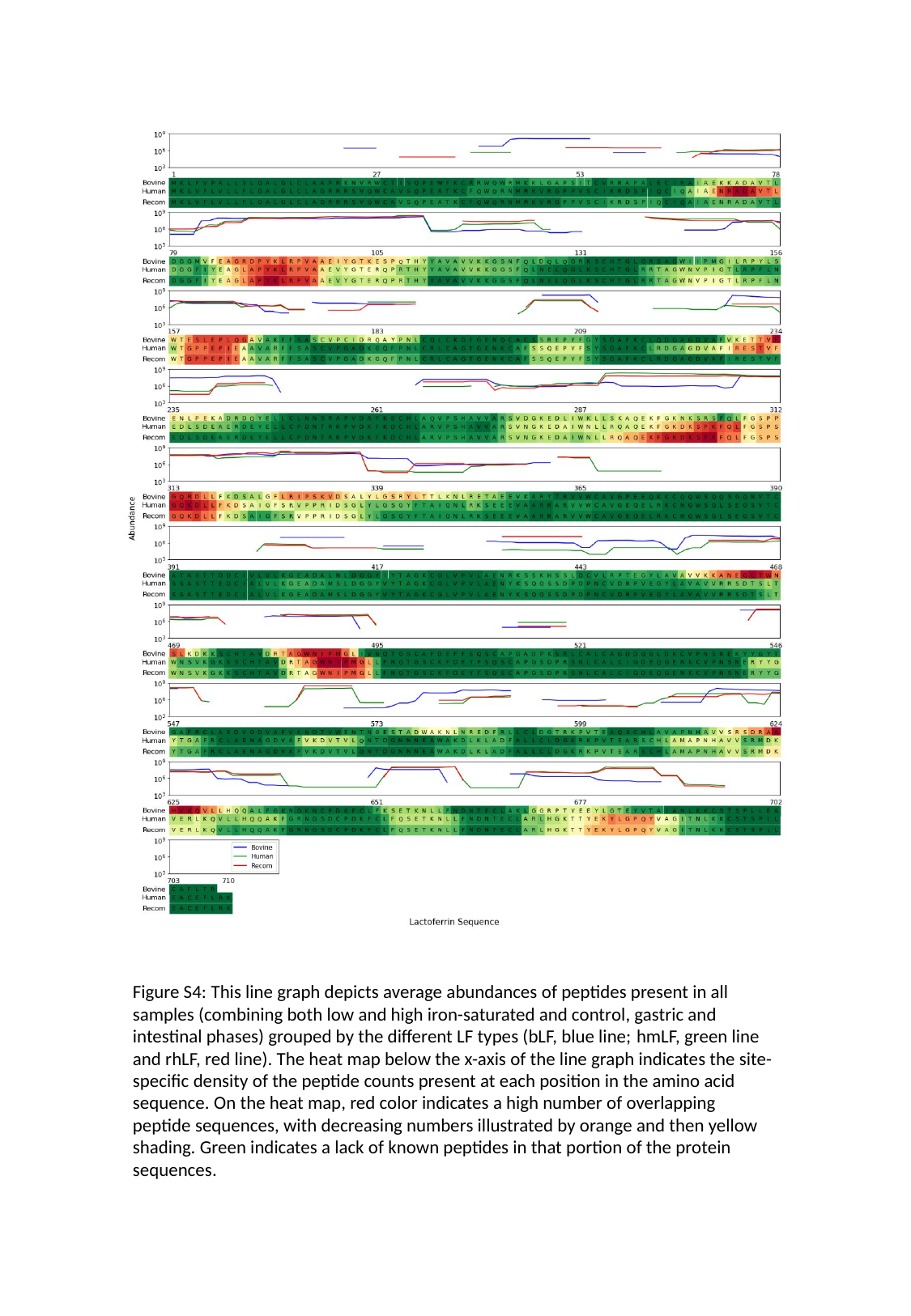

Figure S4: This line graph depicts average abundances of peptides present in all samples (combining both low and high iron-saturated and control, gastric and intestinal phases) grouped by the different LF types (bLF, blue line; hmLF, green line and rhLF, red line). The heat map below the x-axis of the line graph indicates the site-specific density of the peptide counts present at each position in the amino acid sequence. On the heat map, red color indicates a high number of overlapping peptide sequences, with decreasing numbers illustrated by orange and then yellow shading. Green indicates a lack of known peptides in that portion of the protein sequences. ​
