# Supplementary material for: Digestive Profiles of Human Milk, Recombinant Human and Bovine Lactoferrin: Comparing the Retained Intact Protein and Peptide Release
Source: Nutrients. 2024 Jul 21;16(14):2360. doi: 10.3390/nu16142360 (PMC11280017; doi:10.3390/nu16142360)
Supplement: Supplementary file 1 [file nutrients-16-02360-s001.zip › Supplementary tables 1 and 2.pdf]

Table S1. Detection efficiency assessment across human milk, recombinant and bovine lactoferrin samples via ELISA.

Sample ID[1]	Dilution	Lactoferrin type[2]	Spiked protein (ng/ml)	Average of detected protein (ng/mL)	Average without spike (ng/mL)	Detection efficiency (%)[3]
HC_30	40,000	Human	2	2.425	0.923	75.10%
HG_30	10,000	Human	2	1.526	0.1565	68.48%
HI_30	10,000	Human	2	1.586	0.1565	71.48%
HC_83	40,000	Human	2	3.143	1.243	95.00%
HG_83	10,000	Human	2	2.621	1.054	78.35%
HI_83	10,000	Human	2	2.222	0.587	81.75%
RC_44	40,000	Recombinant	2	2.655	0.708	97.35%
RG_44	10,000	Recombinant	2	2.262	1.553	35.45%
RI_44	10,000	Recombinant	2	1.783	0.1565	81.33%
RC_100	40,000	Recombinant	2	2.414	0.627	89.35%
RG_100	10,000	Recombinant	2	2.18	1.128	52.60%
RI_100	10,000	Recombinant	2	1.753	0.1565	79.83%
BC_6	250	Bovine	50	87.263	31.981	110.56%
BG_6	250	Bovine	50	53.48	0.221	106.52%
BI_6	250	Bovine	50	49.031	0.199	97.66%
BC_36	250	Bovine	50	78.386	22.274	112.22%
BG_36	250	Bovine	50	56.812	0.286	113.05%
BI_36	250	Bovine	50	52.751	0.289	104.92%

[1,2]First letter of sample ID represents the sample type: (H, Human milk; R, Recombinant human and B, Bovine).
[1]Second letter of sample ID represents the digestion stage: (C, control (no digestion); G, gastric and I, intestinal).
[1]Ending number of sample ID represents the percentage of iron saturation.
[3]The detection efficiency is the average of two replicates.

Table S2. Measured intact lactoferrin concentrations and calculated relative concentrations of human milk, recombinant and bovine lactoferrin samples.

Sample ID[1]	Lactoferrin type[2]	Digestion phase[3]	Mean ± standard deviation (mg/mL)	Relative mean ± standard deviation (%)
HC_30	Human	Control	5.05 ± 0.40	100 ± 0.00
HG_30	Human	Gastric	0.12 ± 0.00*	2.29 ± 0.00*
HI_30	Human	Intestinal	0.12 ± 0.00*	2.29 ± 0.00*
HC_83	Human	Control	5.60 ± 1.11	100 ± 0.00
HG_83	Human	Gastric	3.50 ± 1.84	62.53 ± 32.85
HI_83	Human	Intestinal	0.46 ± 0.40	8.16 ± 7.13
RC_44	Recombinant	Control	3.75 ± 0.37	100 ± 0.00
RG_44	Recombinant	Gastric	1.11 ± 1.15	29.73 ± 30.76
RI_44	Recombinant	Intestinal	0.12 ± 0.00*	3.09 ± 0.00
RC_100	Recombinant	Control	3.13 ± 0.43	100 ± 0.00
RG_100	Recombinant	Gastric	0.83 ± 0.83	26.39 ± 26.44
RI_100	Recombinant	Intestinal	0.30 ± 0.37	9.66 ± 11.92
BC_6	Bovine	Control	1.01 ± 0.10	100 ± 0.00
BG_6	Bovine	Gastric	0.05 ± 0.00*	5.26 ± 0.00*
BI_6	Bovine	Intestinal	0.05 ± 0.00*	5.26 ± 0.00*
BC_36	Bovine	Control	0.73 ± 0.04	100 ± 0.00
BG_36	Bovine	Gastric	0.05 ± 0.00*	7.31 ± 0.00*
BI_36	Bovine	Intestinal	0.05 ± 0.00*	7.31 ± 0.00*

[1,2]First letter of sample ID represents the sample type: (H, Human milk; R, Recombinant human and B, Bovine).
[1,3]Second letter of sample ID represents the digestion stage: (C, control (no digestion); G, gastric and I, intestinal).
[1]Ending number of sample ID represents the percentage of iron saturation.
*Samples with asterisk fall below the limit of detection for the assay.
